# Super pan-genome reveals extensive genomic variations associated with phenotypic divergence in *Actinidia*

**DOI:** 10.1186/s43897-024-00123-1

**Published:** 2025-01-24

**Authors:** Xiaofen Yu, Minghao Qu, Pan Wu, Miao Zhou, Enhui Lai, Huan Liu, Sumin Guo, Shan Li, Xiaohong Yao, Lei Gao

**Affiliations:** 1 State Key Laboratory of Plant Diversity and Specialty Crops, Wuhan Botanical Garden, Chinese Academy of Sciences, Wuhan, 430074 Hubei China; 2 Hubei Hongshan Laboratory, Wuhan, 430070 Hubei China; 3 University of Chinese Academy of Sciences, Beijing, 100049 China; 4 Bioinformatics Center, College of Plant Science and Technology, Beijing University of Agriculture, Beijing, China

**Keywords:** Kiwifruit, Pan-genome, *Actinidia*, Structural variation, Disease resistant gene

## Abstract

Kiwifruit is an economically and nutritionally important horticultural fruit crop worldwide. The genomic data of several kiwifruit species have been released, providing an unprecedented opportunity for pan-genome analysis to comprehensively investigate the inter- and intra-species genetic diversity and facilitate utilization for kiwifruit breeding. Here, we generated a kiwifruit super pan-genome using 15 high-quality assemblies of eight *Actinidia* species. For gene-based pan-genome, a total of 61,465 gene families were identified, and the softcore and dispensable genes were enriched in biological processes like response to endogenous stimulus, response to hormone and cell wall organization or biogenesis. Then, structural variations (SVs) against *A. chinensis* ‘Donghong’ were identified and then used to construct a graph-based genome. Further population-scale SVs based on resequencing data from 112 individuals of 20 species revealed extensive SVs which probably contributed to the phenotypic diversity among the *Actinidia* species. SV hotspot regions were found contributed to environmental adaptation. Furthermore, we systematically identified resistance gene analogs (RGAs) in the 15 assemblies and generated a pan-RGA dataset to reveal the diversity of genes potentially involved in disease resistance in *Actinidia*. The pan-genomic data obtained here is useful for evolutionary and functional genomic studies in *Actinidia*, and facilitates breeding design.

**Supplementary Information:**

The online version contains supplementary material available at 10.1186/s43897-024-00123-1.

## Core

We present a super pan-genome of eight *Actinidia* (kiwifruit) species. More than one million structural variations were identified among different *Actinidia* species, and SVs potentially involved in the phenotypic divergence and environmental adaptation in *Actinidia* were revealed. In addition, a pan-RGA (resistance gene analog) dataset was created to explore the disease resistance gene reservoir in *Actinidia*. This study offers valuable insights into the genomic dynamics in *Actinidia* and valuable resources for kiwifruit improvement.

## Gene & Accession Numbers

Sequence reads and the genome assembly with annotation were downloaded from China National Genomics Data Center, Figshare database, GitHub and National Center for Biotechnology Information (Table S1, S2, S8, S13).

## Introduction

Kiwifruit, also known as Chinese gooseberry, is an economically and nutritionally important fruit crop enriched with vital nutrients, especially vitamin C. The global kiwifruit production amounted to approximately 4.54 million metric tons (www.statista.com), and was valued at 1.71 billion dollars in 2022. Kiwifruit is a functional dioecious woody vine belonging to the genus *Actinidia* of Actinidiaceae. According to recent revision, there are 54 species and 21 varieties identified in *Actinidia*, which widely distribute throughout eastern Asia ranging from the tropics (latitude 0°) to cold temperate regions (50°N) (Huang 2016). Extensive interspecific hybridizations have driven reticulate speciation and diversification in *Actinidia* (Liu et al. 2017; Yu et al. 2023). Besides, *Actinidia* species exhibit complex patterns of ploidy variations with a high basic chromosome number (x = 29). For example, there are diploids, tetraploids and hexaploids found in *A. chinensis* complex, which comprise the two most commercialized kiwifruit varieties, *A. chinensis* var. *chinensis* and *A. chinensis* var. *deliciosa*. Subdivision of *Actinidia* has long been contentious, and classification based on morphological features was not sustainable and widely acceptable. Li et al. (2000) proposed to divide *Actinidia* into two subgenera, *Leiocarpae* and *Maculatae*. *Leiocarpae*, with smooth-skinned, hairless fruit, was consistently formed a monophyletic group. Nevertheless, *Maculatae* was found polyphyletic which contains individual species varying in frequency of lenticels on the fruit skin and type of leaf hair (Huang 2016). Further subdivision of *Maculatae* was always paradoxical based on morphological and limited molecular evidence. Since genomics-based approaches have proven their power on disentangling elusive phylogenic relationships (Guo et al. 2023; Li et al. 2024a; Wu et al. 2023), these methods should be adopted to elucidate the complicated interspecific relationship within *Actinidia*.

Abundant phenotypic variations were identified among *Actinidia* species. As mentioned above, species in *Leiocarpae* all have glabrous fruit and almost no pubescence on petiole, while *Maculatae* species have spotted fruit and indumentum on small flowering shoot (Huang et al. 1999). Additionally, species in *Maculatae* also have difference in pericarp hair, e.g. *A. eriantha* with long, straight, and bushy trichomes and *A. latifolia* with short, distorted, and spare trichomes. Previous study showed that splicing of Nck-associated protein 1 (NAP1) in *A. latifolia* might be responsible for the shorter trichomes in *A. latifolia* (Miao et al. 2023). However, the genetic basis for pericarp hair variation between *Leiocarpae* and *Maculatae* has not been understood so far. Interestingly, some species in *Leiocarpae*, like *A. arguta*, has a very short softening stage during fruit ripening comparing to species in *Maculatae*, e.g. *A. chinensis* and *A. eriantha* (Lu et al. 2024). Fruit softening involves change of interactions between pectin, xyloglucan and cellulose, and results in breakdown of the cell wall structure. It was reported that galactose loss as well as pectin solubilization started at an earlier firmness stage in *A. arguta* fruits compared to *A. chinensis* var. *deliciosa* fruits (Sutherland et al. 2017). Besides, *A. eriantha* and *A. latifolia* contain higher vitamin C content in fruits than other species (Huang et al. 2000), and the expression level of *GGP3* gene was associated with ascorbic acid (AsA) content in *A. eriantha* (Liu et al. 2021, 2022). A recent study showed that duplication of *ERF098* transcription factor in *A. latifolia* and *A. eriantha* might account for AsA biosynthesis and accumulation in their fruits (Han et al. 2023). These studies expounded the genetic basis for phenotypic variants in one or two *Actinidia* speices, while few studies paid attentions to structural variations (SVs) or presence/absence variants (PAVs), which were found responsible for many phenotypic variations in other species (Qin et al. 2021; Lyu et al. 2023).

It is worth mentioning that, comparing to the cultivated kiwifruits, some wild relatives are more resistant to disease (Wang et al. 2019; Wang et al. 2020a). Kiwifruit industry is threatened by many severe diseases, including bacterial canker, ripe rot, black spot, gray mold and so on (Erper et al. 2013; Pereira et al. 2021; Li et al. 2022; Yang et al. 2022; Zhao et al. 2023). Among them, kiwifruit bacterial canker, caused by *Pseudomonas syringae* pv. *actinidiae* (*Psa*), has posed a serious threat to global kiwifruit industry. *Psa* damages both *A. chinensis* var. *chinensis* and *A. chinensis* var. *deliciosa*, resulting in severe economic losses (Pereira et al. 2021). Evaluation on the resistance to *Psa* in wild *Actinidia* germplasm showed that some accessions from *A. eriantha* and several species in *Leiocarpae*, like *A. valvata*, *A. arguta*, and *A. ploygama*, were highly resistant to *Psa*, indicating the potential presence of bacterial canker resistant genes in these *Actinidia* germplasm (Michelotti et al. 2018; Song et al. 2019; Wang et al. 2019; Wang et al. 2020a). A major way in plant disease resistance breeding is to introduce resistant genes to the susceptible cultivars, therefore, a survey of plant resistance gene analogs (RGAs) in the wild relatives will lay a basic foundation for improving plant resistance to disease (Tirnaz et al. 2020; Amas et al. 2023).

With the rapid development of high-throughput sequencing technologies and genomic-based methods, genomes from several *Actinidia* species and varieties have been sequenced and assembled. The first draft genome assembly of *Actinidia* (*A. chinensis* ‘Hongyang’) was published at 10 years ago (Huang et al. 2013). Since then, many kiwifruit genomes have been reported, including those of *A. arguta*, *A. chinensis*, *A. eriantha*, *A.hemsleyana*, *A. latifolia*, *A. ploygama*, *A. rufa*, and *A. zhejiangensis* (Pilkington et al. 2018; Tang et al. 2019; Wu et al. 2019; Tahir et al. 2022; Yao et al. 2022; Akagi et al. 2023; Han et al. 2023; Wang et al. 2023b; Xia et al. 2023; Yu et al. 2023; Yue et al. 2023; Lu et al. 2024;Yue et al. 2024; Zhang et al. 2024). Some of them achieved telomere-to-telomere (T2T) and gap-free level (Han et al. 2023; Wang et al. 2023b; Yue et al. 2023). Pan-genome can capture genetic diversity from different individuals or populations and reveals genomic complexity (Liu et al. 2020; Qin et al. 2021; Shang et al. 2022; Lyu et al. 2023; Yan et al. 2023). The first kiwifruit pan-genome for *A. chinensis* was recently generated, and a novel SV mediating fruit coloration and fruit quality was found, providing valuable information for kiwifruit genomics-assisted breeding (Wang et al. 2024). More recently, a primary gene-based pan-genome of different *Actinidia* speices has been constructed and facilitated the identification of a gene related to high vitamin C content (Li et al. 2024b). Although many kiwifruit genomes and resequencing data have been published, interspecific genetic diversity among *Actinidia* species has not been comprehensively investigated. In this study, we took advantage of 15 high quality assemblies from eight *Actinidia* species to build a super pan-genome to explore the genetic diversity among *Actinidia*. Together with resequencing data from 112 individuals of 20 species, we systematically identified SVs potentially contributed the phenotypic variations in *Actinidia*. Meanwhile, we investigated the interspecific relationship within *Actinidia*, and potential genetic architecture driving the divergence of *Leiocarpae* and *Maculatae*. Finally, we generated pan-RGA to explore the disease resistance gene resources in *Actinidia*, providing a basic understanding of resistance genes to target for genomics-based improvement breeding.

## Results

### Genome assemblies and their phylogenic relationship

**Fig. 1:**
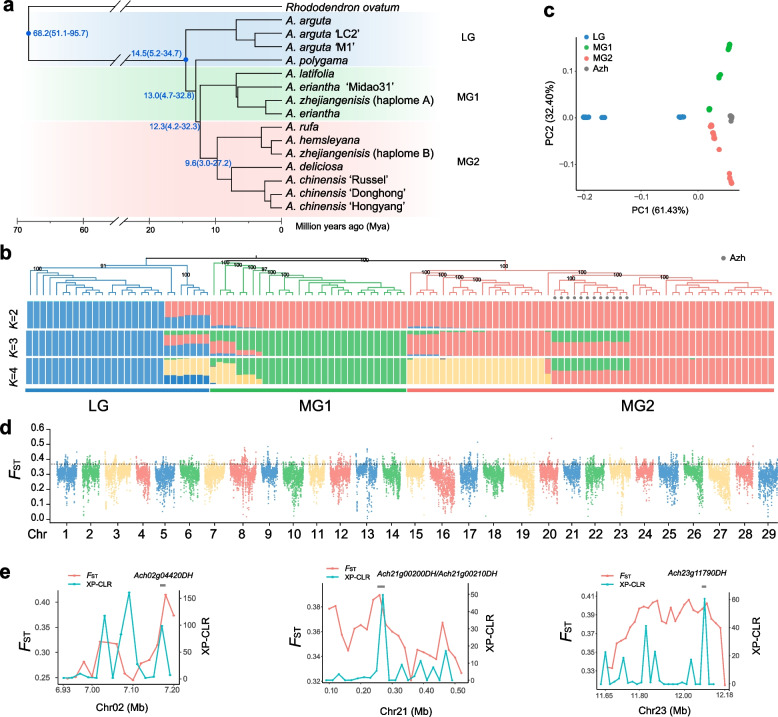
The phylogenetic relationship and genetic variations within *Actinidia*. **a** Phylogenic tree of *Actinidia* species based on all orthologous genes. Blue dots on the tree node represent calibration times obtained from the TimeTree database (http://www.timetree.org/). **b** Maximum-likelihood phylogenetic tree and population structure of *Actinidia* individuals based on SNPs. Azh: *A. zhejiangenisis* (**c**) Principal component analysis plot showing the first two components and the percentage of variation explained by each component. **d** *F*_ST_ analysis across the 29 chromosomes between LG and MG. Black dotted line represent the threshold line (95th percentile). **e** XP-CLR and *F*_ST_ analysis of particular regions

To construct a super pan-genome of *Actinidia* species, we collected 15 published high-quality kiwifruit genome assemblies from eight *Actinidia* species (Table S1). Among them, four assemblies were from the *Leiocarpae* group, others were from *Maculatae* group. These assemblies had total lengths ranging from 608.3 to 652.8 Mb and BUSCO scores between 93.0 and 99.3% with an average of 97.7%, indicating their high completeness. All the assemblies were annotated according to the pipeline used in our previous study (Yu et al. 2023), resulting in gene number ranging from 40,311 to 46,308 with an average BUSCO value of 94.2% for the 15 assemblies. Orthologue genes were identified and a species tree was inferred from all sets of orthogroup genes (Fig. 1a). The analyzed *Actinidia* species could be divided into two groups, i.e. *Leiocarpae* group (LG) and *Maculatae* group (MG). LG contained *A. arguta* and *A. ploygama,* MG contained *A. chinensis*, *A. eriantha*, *A. hemsleyana*, *A. latifolia*, *A. rufa* and *A. zhejiangensis.* Molecular dating indicated that MG diverged from LG about 13.0 million years ago (Mya). The MG consisted of two separate clades, named MG1 and MG2, and they diverged from each other about 12.3 Mya (Fig. 1a). The two haplomes of *A. zhejiangensis* were assigned into MG1 and MG2, separately, aligned with our previous finding of the hybrid origin of *A. zhejiangenisis* (Yu et al. 2023).

Due to the sparse kiwifruit species with genome assemblies available, the deduced phylogenic relationship can only cover limited diversity of the *Actinidia* genus. To address this problem, we further collected resequencing data of 114 *Actinidia* accessions (28 LG and 86 MG individuals) from 20 species (Table S2). Reads were mapped to the *A. chinensis* cv. Donghong (hereafter AcDH) genome and then used for SNP calling (Fig. 1b). Phylogenetic tree inferred from whole-genome SNPs supported the dividing of LG and MG within *Actinidia*, and separation of MG1 and MG2 within MG (Fig. 1b), resembling the species tree based on orthologous genes (Fig. 1a). Beside *A. arguta* and *A. ploygama*, the LG also contained *A. macrosperma* and *A. valvata*, the MG1 contained *A. latifolia*, *A. eriantha*, *A. cylindrica*, *A. lanceolata* and so on, the MG2 contained *A. chinensis*, *A. hemsleyana*, *A. rufa*, *A. setosa*, etc. The admixture analyses showed that most LG, MG1 and MG2 had distinct compositions when *K* = 3, and a few individuals had mixed compositions. For example, the known hybrid *A. zhejiangenisis* had a mixed composition originated from MG1 and MG2, respectively. Principal component analysis (PCA) also suggested that these accessions could be clearly divided into three clusters except *A. zhejiangenisis*, which was placed between MG1 and MG2 (Fig. 1c). All these results suggested that MG diverged in two separate clades after diverging from LG.

To further detect the genetic architecture driving the divergence of LG and MG, the pairwise genetic differentiation (*F*_ST_) between the LG and MG individuals was evaluated. Regions with *F*_ST_ value above the 95th percentile were identified as highly differentiated regions (HDRs). A total of 68.08 Mb HDRs were identified between the LG and MG, and the top three chromosomes with the longest HDRs between LG and MG were Chr08, Chr23 and Chr13 (Fig. 1d, Table S3). The HDRs contained 4,334 genes, and Gene Ontology (GO) enrichment analysis showed these genes were significantly enriched in biological processes including cellular response to stress, terpenoid metabolic process and plant-type secondary cell wall biogenesis (Fig. S1). To detect select sweep in MG after divergence from LG, we scanned genomic regions with extreme allele frequency differentiation over extended linked regions using XP-CLR. A total of 43.91 Mb regions were selected in MG overlapping with 3,900 genes (Table S4), including 18 genes participated in cell wall organization or biogenesis, such as expansin gene and xyloglucan fucosyltransferase gene. Besides, a total of 8.33 Mb regions were found in both the HDR and XP-CLR selected region, overlapping 517 genes, including some trichome initiation or development genes, like *Ach02g04420DH*, a trichome birefringence-like gene (Kabir et al. 2023), *Ach23g11790DH*, a MYB-like transcription factor homologous to *ETC1* (Wang et al. 2008), and some genes participated in cell wall organization, like *Ach21g00200DH* and *Ach21g00210DH* encoding cellulose synthase A catalytic subunit (Fig. 1e, Table S5). Species in LG have glabrous and smooth fruit skin, while those in MG have spotted or hairy fruit skin (Huang 2016). The HDRs region and selected regions may contribute to underlie genetic basis for phenotypic variations between LG and MG.

### Gene-based *pan*-genome of Actinidia

**Fig. 2:**
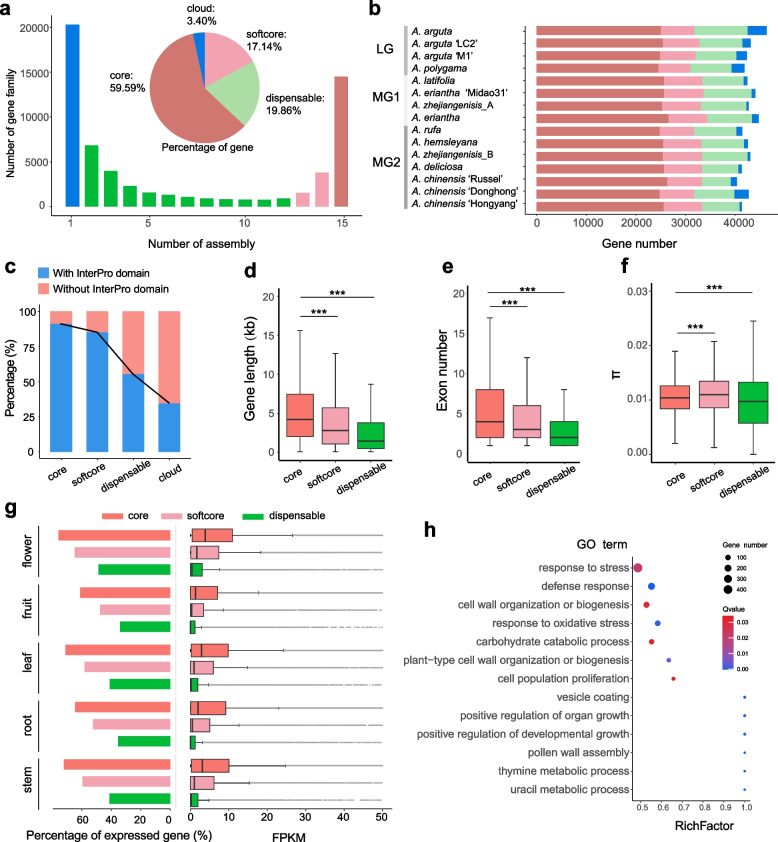
Gene-based pan-genome of *Actinidia*. (**a**) Composition of the pan-genome. The histogram shows the number of gene families in the 15 assemblies with different frequencies. Pie shows the proportion of core, softcore, dispensable, and cloud genes. **b** The core, softcore, dispensable, and cloud gene numbers in each assembly. **c** Proportions of genes with and without InterPro domains in core, soft core, dispensable, and cloud genes. The distribution of gene lengths (**d**), exon numbers (**e**) and nucleotide diversity (**f**) of core, softcore and dispensable genes. ***p < 0.001, Student’s *t* test. **g** Expression levels of core, softcore and dispensable genes in *A. eriantha* at different tissues. **h** GO enrichment analysis of softcore and dispensable genes

The 15 kiwifruit assemblies included four LG, four MG1 and seven MG2 assemblies and were obtained five male and nine female individuals (Table S1). Ortholog investigation classified all genes into 61,465 families comprising 14,492 core (present in all 15 assemblies), 5,347 softcore (present in all 13–14 assemblies, over 85% assemblies), 21,326 dispensable (present in all 2–12 assemblies), and 20,300 cloud (present in only one assembly) gene families (Fig. 2a). Although dispensable and cloud gene families accounted for a larger proportion (67.72%) of the pan-genome, as the average numbers of genes belonging them were much less than those of core and softcore gene families (Fig. S2), they occupied less than a quarter of total genes. Each assembly had an average of 3.37% cloud genes (Fig. 2a, 2b). Meanwhile, core gene families contained over half of the total genes, and at least 54.09% for each assembly.

We found that 91.10% of the core genes, and 85.07% of the softcore genes contained InterPro domains, which were much higher than the percentages in the dispensable and cloud genes (55.37% and 34.57%, respectively) (Fig. 2c). Besides, the average length and exon number of core genes were significantly longer than other groups of genes (Fig. 2d, 2e), indicating that gene structure was more complex in core genes than softcore and dispensable genes. Meanwhile, core genes had lower nucleotide diversity (π) in their coding sequences than softcore genes, but higher than the dispensable genes (Fig. 2f), suggesting that the core genes were more functionally conserved than softcore genes. Additionally, core genes had both higher percentage of expressed genes (fragments per kilobase of exon model per million mapped fragments (FPKM) > 0.5) and higher expression levels than softcore and dispensable genes in different tissues (Fig. 2g, Fig. S3), GO enrichment analysis showed that core genes were enriched for several essential biological processes, including nitrogen compound and phosphorus metabolic processes, RNA processing, chromosome organization and cellular response to DNA damage stimulus (Fig. S4), while the softcore and dispensable genes were enriched in response to stress, defense response, response to oxidative stress, and cell wall organization or biogenesis etc. (Fig. 2h).

A total of 74 gene families (containing 361 genes) were found in each LG but not in any MG genome, that is, specific to LG, including several gene families involved in cell wall organization, like genes encoding trichome birefringence-like protein or glycoside hydrolase (Table S6). These specific gene families probably contribute to the fruit characteristics, like smooth and hairless fruit skins of LG. Meanwhile, 140 gene families (containing 608 genes) were specific to MG1, including gene families encoding redox enzymes, like cytochrome P450, NAD-dependent epimerase and ferredoxin reductase, and one gene family encoding hydroxyacylglutathione hydrolase, which plays vital role in glutathione (GSH) metabolism (Dorion et al. 2021). Previous study reported that GSH participated in the AsA metabolism in *A. eriantha* via AsA-GSH cycle (Liao et al. 2021), thus this MG1-specific gene families might play a role in the AsA metabolism in *A. latifolia* and *A. eriantha*, which had high vitamin C content in fruits. Besides, gene families specific to male accessions included a gene family homologous to *FrBy*, a known *Actinidia* sex-determining genes (Akagi et al. 2019), and two gene families with unknown function, which didn’t not locate in the known *Actinidia* sex-determining region (Table S7), suggesting this gene family was probably not related to sex differentiation. Meanwhile, no gene families specific to female accessions were found.

### Global landscape of structural variations in *Actinidia*

**Fig. 3:**
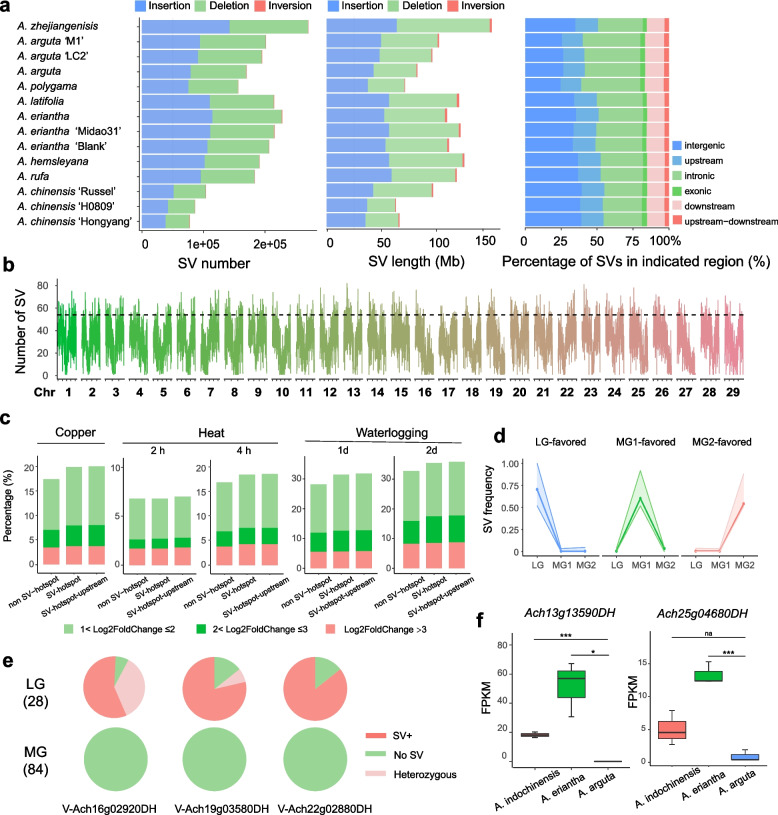
Structural variations and graph-based pan-genome of *Actinidia*. **a** The SV numbers, lengths and percentage of SVs in indicated regions in each accession. **b** SV numbers on chromosomes in 200 kb windows. Regions over the threshold line (95th percentile) are SV-hotspot regions. **c** The percentage of DEGs in SV-hotspot genes, SV-hotspot-upstream genes and non-SV-hotspot genes under copper, heat and waterlogging stresses. **d** SV frequency of LG-, MG1-, and MG2-favored SVs. The broken lines indicate the averaged frequencies, and the ribbons indicate the ranges of SV frequencies. **e** Genotypes of three SVs in LG and MG groups. (**f**) Expression levels of *Ach13g13590DH* and *Ach25g04680DH* in the fruit peels from three kiwifruit species. ***p < 0.001, *p < 0.05, Student’s *t* test

To identify SVs, both genome assemblies and third-generation sequencing (TGS) data of 11 accessions were aligned against the AcDH genome by PanPop (Table S8), a tool that can enhanced SV accuracy by merging and filtering of SVs from multiple SV callers (Zheng et al. 2024). As for *A. zhejiangenisis* and another two *Actinidia* accessions without public assemblies (*A. chinensis* 'H0809' and *A. eriantha* 'Blank'), SVs were investigated using only TGS data. Thus, SVs in 14 *Actinidia* accessions from eight species were identified. In short, except *A. zhejiangenisis*, we identified an average of 164,777 SVs (≥ 50 bp in size) per accession (ranging from 77,536 SVs for *A. chinensis* 'Hongyang' to 228,098 for *A. eriantha*), affecting an average of 96.54 Mb of sequence per accession (ranging from 63.16 Mb for *A. chinensis* 'H0809' to 125.44 Mb for *A. hemsleyana*) (Fig. 3a). *A. zhejiangenisis*, which contained genetic information of two parent species resulting the largest number of SVs (270,419). Insertions and deletions accounted for the majority of SVs, and inversions were much less than insertions and deletions. If excluding *A. zhejiangenisis*, no significant difference in SV numbers or total lengths were found between LG and MG1, or MG1 and MG2 accessions. Most SVs located in intergenic and intronic regions, then upstream and downstream of genes. All the SVs were merged to generate a graph-based genome with a set of 1,277,140 non-redundant variations.

We further mapped the resequencing short reads of the 112 *Actinidia* accessions (Table S2) onto the graph-based pan-genome. After filtering, a total of 162,866 variations were obtained, including 80,803 insertions and 82,063 deletions. The NJ tree based on the SVs showed these individuals could be classified into three groups: LG, MG1 and MG2, and similar result was found in PCA analysis (Fig. S5) in line with the SNP analyses (Fig. 1c). Besides, we found an uneven distribution of SVs along the chromosomes, and 532 SV hotspot regions were identified (Fig. 3b, Table S9). The top three chromosomes with longest SV hotspot regions were Chr15 (4.16 Mb), Chr20 (4.12 Mb), Chr08 and Chr03 (4.02 Mb), that is, the most dynamic chromosomes. GO enrichment analysis showed that the genes in SV hotspot regions were significantly enriched in plant organ formation, which might be associated with the phenotypic variations among different *Actinidia* species, and regulation of gene expression (Fig S6). We therefore explored the general expression characterization of SV-hotspot genes (genes with gene sequences overlapping with SV-hotspot regions), SV-hotspot-upstream genes (genes with 2 kb upstream overlapping with SV-hotspot regions) and non-SV-hotspot genes (neither SV-hotspot, nor SV-hotspot-upstream genes) using RNA-seq data of *A. chinensis* under different abiotic stress conditions including copper, heat and waterlogging stresses (Fig. 3c). The percentage of significantly differentially expressed genes (DEGs) were calculated in the three types of genes, and we found that both the SV-hotspot and SV-hotspot-upstream genes had higher percentages of DEGs than non-SV-hotspot genes. Moreover, the SV-hotspot or SV-hotspot-upstream genes had obviously higher proportion of genes with > fourfold expression changes after stress treatment than the proportion of non-SV-hotspot genes, indicating that SV-hotspot and SV-hotspot-upstream are generally more sensitive to environmental stresses. These results suggesting SV hotspot regions may undergo stronger environmental selection compared to other genome regions, which was consistent with the previous findings in rice and *Malus* accessions (Qin et al. 2021; Wang et al. 2023a).

The frequency of each SV in LG, MG1 and MG2 individuals except *A. zhejiangenisis* was calculated, respectively, and LG-favored SVs with high frequency (> 0.5) in LG but low frequency (< 0.05) in MG1 and MG2 individuals, were identified, as well as MG1- and MG2-favored SVs. A total of 18,364 LG-favored, 995 MG1-favored and 3,743 MG2-favored SVs were identified (Fig. 3d). Among the LG-favored SVs, nearly three quarters of SVs only existed in LG individuals, and these SVs overlapping with the genes enriched in various biological processes, such as nitrogen compound metabolic process, phosphorus metabolic process and cellular response to stimulus (Fig S7). It's worth noting that some of them were related to trichome development. For example, a 55-bp insertion in the exon of *Ach22g02880DH*, a gene homologous to *AtPIR* gene, which played role in actin filament reorganization and trichome development (Isner et al. 2017); a 114-bp deletion in the second intron of *Ach19g03580DH*, a gene homologues to *WER*, which encodes a MYB transcript factor regulating non-hair cell fate (Lee et al. 1999), a 114-bp insertion in the last exon of *Ach16g02920DH*, a gene encoding polygalacturonase, were found present in most LG individuals, but absent in MG (Fig. 3e, Table S10). Besides, 4,784 LG-favored SVs located in the promoter regions of 5,230 genes. For instance, a 218-bp insertion on the promoter of *Ach13g13590DH*, a gene homologous to *CPC*, and a 205-bp insertion on the promoter of *Ach25g04680DH*, a gene homologous to *GL3*. Both *CPC* and *GL3* were major regulators of hair cell fate establishment (Wang et al. 2022). We then compared the expression levels of *Ach13g13590DH* and *Ach25g04680DH* in the fruit peels from three kiwifruit species, *A. arguta* in LG with hairless fruit skin, *A. eriantha* in MG1 hairy fruit skin, and *A. indochinensis* in MG2 with spotted fruit skins. Their expression levels in *A. arguta* were significantly lower than that in *A. eriantha* or *A. indochinensis* (Fig. 3f), suggesting that these two genes might contributed to the diversity of fruit skin of *Actinidia*.

The MG1-favored and MG2-favored SVs overlapped with 263 and 1,431 genes, respectively. These genes participated in various biological processes, including establishment of localization, cellular response to stimulus, regulation of biological process, cell wall organization or biogenesis and so on (Table S11). Besides, we found 21.48% of all the favored SVs located in the SV-hotspot regions. Overall, those SVs favored or specific in given group might contributed to the phenotypic diversity or environmental adaptation of *Actinidia*.

### Disease resistance genes reservoir and *Pan*-RGA of Actinidia

**Fig. 4:**
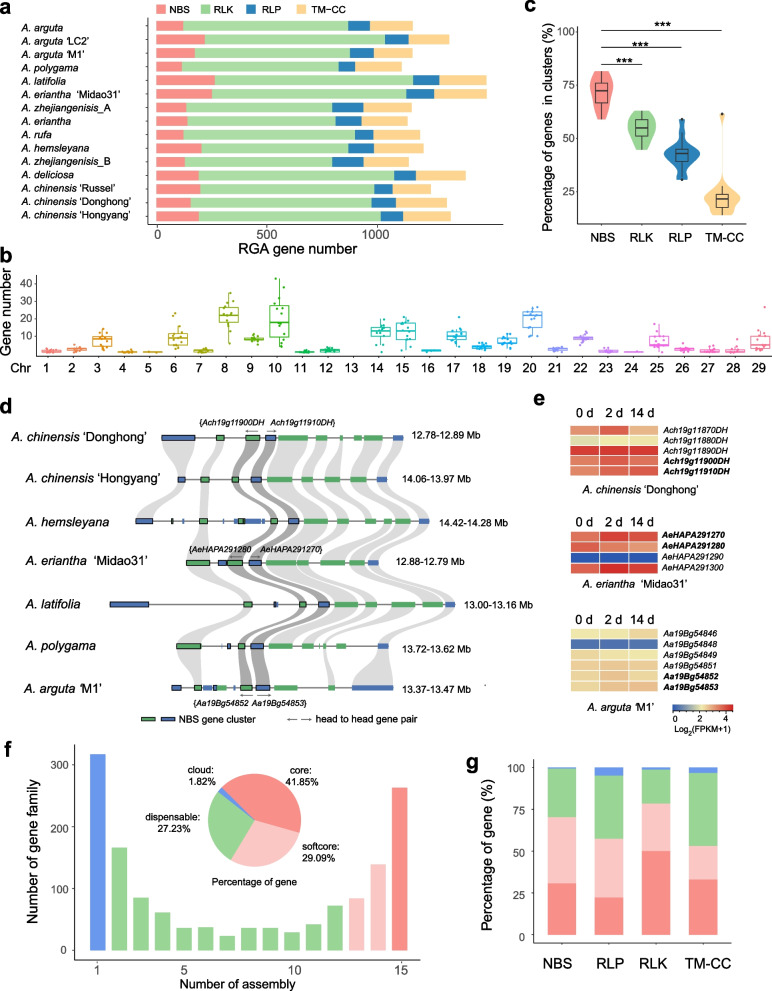
Resistance gene analogs (RGAs) in the 15 *Actinidia* assemblies. **a** RGA gene numbers in each assembly. **b** NBS gene number on each chromosome. **c** The percentage of genes located in clusters of each type. **d** A representative NBS cluster on chromosome 19 has conserved paired NLRs among the *Actinidia* accessions, and seven representative accessions are used here for displaying. **e** Expression levels of genes in the NBS cluster after *Psa* infection. Three representative accessions are used here. **f** Composition of the pan-RGA. The histogram shows the number of gene families in the 15 assemblies with different frequencies. Pie shows the proportion of core, softcore, dispensable, and cloud RGA genes. **g** Percentages of core, softcore, dispensable, and cloud genes in each type of RGAs

RGAs are potential *R* genes with specific conserved domains and motifs. To explore the reservoir of disease resistance genes in *Actinidia* species, we identified all classes of RGAs from the 15 *Actinidia* assemblies (Table S1). In total, 18,858 RGAs were detected, including 2,626 nucleotide-binding site (NBS)-encoding proteins, 11,495 receptor-like protein kinases (RLKs), 1,624 receptor-like proteins (RLPs) and 3,113 proteins with both transmembrane domain and coiled-coil domain (TM-CCs) (Fig. 4a). The identified RGAs in each assembly ranging from 1,110 to 1,494, and the difference in RGA numbers was not only observed between species, but also within species. RLKs accounted for the largest portion of RGAs in each assembly, and almost randomly distributed on the 29 chromosomes (Fig. S8). Meanwhile, NBS genes exhibited very uneven distribution pattern on chromosomes comparing to other types of RGAs, with Chr08, Chr10 and Chr20 containing significantly higher numbers of NBS genes than other chromosomes (Fig. 4b). These RGAs, combined with molecular markers from known disease resistance QTL, could be used to identify candidate resistance genes. For example, we found some RLKs in the previous identified QTL regions for *Psa* resistance on Chr27 (Tahir et al. 2019). Among them, one RLK gene, *Ach27g03710DH*, displaying presence/absence variations (PAVs) among different species, might contribution to response to *Psa* infection.

The genes belonging to same family and within 200 kb of each other in the genome were taken as gene clusters (Van de Weyer et al. 2019). We found most NBS genes in each assembly tend to locate in such clusters, and the percentage of clustered genes in NBS genes was significantly higher than those in RLKs, RLPs and TM-CCs (Fig. 4c). The NBS gene cluster mainly constituted of 2–5 genes, and the largest cluster in each assembly constituted of 6–15 genes (Fig. S9). Paired nucleotide-binding leucine-rich repeat (NLR) genes, a particularly interesting subset of NBS genes in head-to-head orientation (van Wersch and Li 2019), were identified. Each accession had an average of 6.77% paired NLRs of NBS genes, and we found one pair of paired NLR genes evolutionarily conserved in all analyzed *Actinidia* accessions (Fig. 4d). In AcDH, this gene pair, *Ach19g11900DH* and *Ach19g11910DH*, located in a NBS cluster of five genes on chromosome 19. We further explored the expression levels of genes in this NBS cluster after *Psa* infection in AcDH, *A. eriantha* ‘Midao31’ and *A. arguta* ‘M1’. The genes in this cluster exhibited inconsistent expression patterns at three stages, whereas the two genes of the paired NLR showed similar expression levels in all stages (Fig. 4e), suggesting that this paired NLR probably not only evolutionarily conserved, but also functionally linked for *Actinidia* defense response.

Integrated domains (IDs) of NBS genes, which participate in interaction with effectors, were identified. The top three most frequent IDs of NBS were AAA ATPase, C-JID and NACHT domains (Fig. S10), which were shared by all accessions. Meanwhile, different accessions exhibited different IDs compositions, for instance, only the three conserved ID domains were shared by the three *A. arguta* accessions. Additionally, 17, 22 and 28 ID domains were found in different *A. chinensis*, *A. eriantha* and *A. arguta* accessions, respectively (Fig. S11). Besides, 10, 15 and 18 IDs were unique to LG, MG1 and MG2, respectively. The diversity of ID domain distribution within and among *Actinidia* species may be associated to their different performance of disease resistance.

Multiple mutants were found on the RGA genes, indicating the abundant genetic diversity of RGAs in *Actinidia*. We further built a pan-RGA of *Actinidia* species to reveal the diversity of disease resistance genes. These RGAs were grouped into 1,426 gene families based on sequence similarity, including 263 core (present in all 15 assemblies), 223 softcore (present in all 13–14 assemblies), 623 (present in all 2–12 assemblies) and 317 cloud gene families (present in only one assembly) (Fig. 4f). At gene level, the pan-RGA comprised 7,893 core, 5,486 softcore and 5,136 dispensable genes, accounted for 41.85%, 29.09%, and 27.23% of all RGAs, respectively. Comparing to the whole pan-genome, pan-RGA had much lower percentage of core genes, but more percentage of softcore genes. Additionally, RLK genes had higher percentages of core genes than other type genes (Fig. 4g). For each assembly, core, softcore and dispensable genes had a slight difference in number (Fig. S12). Besides, we found 184 dispensable gene families (575 genes) absent in *A. chinensis*, the most commercialized kiwifruit species, and 28 dispensable gene families (67 genes) absent in MG (Table S12). These genes may be valuable resource for future resistance breeding of kiwifruit.

## Discussion

*Actinidia* was previously proposed to be divided into *Leiocarpae* and *Maculatae* group based on morphological evidences (Li et al. 2000), while the further classification of *Maculatae* was not sustainable (Huang 2016). Classification based on morphological evidence has some limitations as some species exhibit abundant morphological variations. Therefore, here we explored the evolutionary relationships within *Actinidia* by genomic approaches. We generated phylogenic trees based on ortholog genes, SNPs and SVs (Fig. 1 and Fig. S5, S14), and all the results showed that *Maculatae* group could be subdivided into two subgroups. Huang et al. (1999) suggested dividing *Actinidia* into three sections: *Leiocarpae*, members in this section have smooth-skinned fruits; *Maculatae* includes the species with spotted fruits, and *Vestitae* has leaf hairs base. Nevertheless, the MG1 and MG2 groups did not correspond to *Maculatae* and *Vestitae* groups suggested by Huang et al. (1999). Moreover, no common distinguishable phenotypic difference between MG1 and MG2 was observed in terms of the reported 50 morphological characters of *Actinidia* (Huang et al. 1999), which may be due to ongoing interspecific hybridization and gene flow between MG1 and MG2 (Liu et al. 2017; Yu et al. 2023). Yet we could not rule out the presence of the distinguishable phenotypes between MG1 and MG2 beyond the scope of previous studies.

Besides, we noticed that the phylogenic tree based on single copy gene had a minor difference at the topology of MG2 with the trees based on all orthologs. Specifically speaking, *A. chinensis* var. *deliciosa* was outside of the *A. chinensis* var. *chinensis* and *A. rufa* branches in the tree based on single copy genes (Fig S13), while closely related to *A. chinensis* var. *chinensis* in the tree based on all orthologs (Fig. 1a). This could be caused by the interspecific gene flow among the MG2, which was mentioned in other study (Liu et al. 2017), or the incomplete lineage sorting (ILS) of ancestral polymorphisms. Moreover, the *A. chinensis* var. *deliciosa* individuals did have mixed component in the admixture analysis when *K* = 3, suggested potential interspecies hybridization between *A. chinensis* and other *Actinidia* species.

Fruit hair is an important appearance quality affecting the market value of kiwifruits. *Leiocarpae* group has smooth-skinned, hairless fruit, such as *A. arguta* with edible and hairless fruit skin; while *Maculatae* group has spotted or hairy fruit skin, such *A. chinensis* var. *deliciosa* with rough and hairy skins, which is not convenient for direct consumption. In this study, we tried to uncover the genetic basis for phenotypic variations of fruit skin between *Leiocarpae* and *Maculatae* groups using different approaches. In short, we found a number of genes associated with trichome initiation or development and plant-type cell wall organization might contribute to the fruit skins variations in *Actinidia*, like trichome birefringence-like genes, some genes for MYB transcription factors, pectinesterase or polygalacturonase and so on. Trichome development can be divided into six stages, and the last stage of its development is maturation of the cell wall (Kubátová et al. 2019). The secondary cell wall consists of the outer, cellulose-rich layer and the inner, callose-rich layer for Arabidopsis trichomes (Kulich et al. 2015). As genes related to trichome development and plant-type cell wall organization were found associated with the phenotypic diversity in *Actinidia*, Thus, we speculated that *Actinidia* species might have diverse secondary cell wall components of trichomes. On the other hand, it was found that *A. arguta* fruits soften quickly and show a much shorter shelf life than *A. chinensis*, which might due to the different expression levels of *CEL1*, a gene encoding cellulose, and *PME1*, a gene encoding pectin methylesterase (Lu et al. 2024). Therefore, the differentiated cell wall biogenesis or organization related genes might not only have roles in trichome biogenesis and development, but also affect fruit ripening or shelf life between *Leiocarpae* and *Maculatae* groups.

Increasing evidence showed that SVs are responsible for many phenotypic variations (Qin et al. 2021; Lyu et al. 2023; Yan et al. 2023; Wang et al. 2023a). Here, we generated a graph-based pan-genome integrating SVs from 15 *Actinidia* accessions, and further identified SVs based on the pan-genome at population scale (Fig. 3). Our result showed SVs can also support inferences about the evolutionary relationships as SNPs. Moreover, SV hotspot regions, the most dynamic genomic region in *Actinidia*, contributed to responses to environmental pressures, as SV-hotspot and SV-hotspot-upstream genes were more sensitive to abiotic stresses than genes in other regions. *Actinidia* has a wide distribution in eastern Asia, and different groups of *Actinidia* taxa had characteristic geographic distributions, e.g., the *Leiocarpae* were to be found mainly in north China with relative cold and dry condition (Huang et al. 2016). Thus, the dynamic genomic variations might contribute to environmental adaptation of *Actinidia* (Zhang et al. 2023). Besides, some SVs were found selected in particular group, and many LG-favored SVs altered gene sequences or regulatory sequences of genes participating in trichome development or cell wall organization. Therefore, SVs might regulate the phenotypic diversity of *Actinidia* and drive the differentiation of MG and LG by changing gene structure or expression of nearby genes. Our study analyzed a small *Actinidia* population, and further studies should concentrate on SVs in larger *Actinidia* population along with accurate phenotypic or environmental data, which will allow SV-based association studies for trait associated gene discovery and improvement breeding.

The sustainable control of plant pathogens relies on the application of genetic resistance primarily driven by RGAs. Under the guidance of the gene-based pan-genome, we systematically identified all the RGAs across the 15 *Actinidia* assemblies and have generated a pan-RGA dataset for *Actinidia* (Fig. 4). Although some studies had identified disease genes in *A. chinensis* and *A. eriantha* (Wang et al. 2020b; Yao et al. 2022), we firstly revealed RGA diversity in *Actinidia* on a large scale covering the LG and MG. We found most NBS genes in clusters, and one paired NLR evolutionarily conserved in *Actinidia*, exhibited similar expression levels after *Psa* infection. Besides, a total of 205 gene families from pan-RGA were found absent in all *A. chinensis* assemblies. These genes greatly enriched the disease resistance gene reservoir for future resistance breeding of kiwifruits. The pan-RGA dataset could not only reveals the disease gene diversity in *Actinidia*, but also be used to identify candidate resistant genes combined with molecular markers from known disease resistance QTL.

In conclusion, the pan-genome construction and genetic variation identification in this study comprehensively reveal the genetic diversity of *Actinidia* species, which will greatly benefit the *Actinidia* breeding and functional genomics research.

## Methods

### Genome sequences collection and annotation

Previously published 15 genome assemblies of eight *Actinidia* species, including *A. arguta*, *A. chinensis*, *A. eriantha*, *A.hemsleyana*, *A. latifolia*, *A. ploygama*, *A. rufa*, and *A. zhejiangensis*, as well as an outgroup, *R. ovatum* genome were downloaded from China National Genomics Data Center, Figshare database, GitHub and National Center for Biotechnology Information (NCBI) (Table S1). As *A. zhejiangensis* was demonstrated as F_1_ hybrid of *A. eriantha* and *A.hemsleyana* in previous study (Yu et al. 2023), the two haplomes with different origins were taken as two assemblies in this study. For other haplotype-resolved genomes, the haplome with the longest sequence or most gene number was chosen as representative (Table S1). BUSCO analysis (v5.0) was performed with the ‘eudicots_odb10’ database to evaluate genome/gene completeness (Manni et al. 2021). All the other assemblies were aligned to the T2T genome of AcDH to check the chromosome order and direction by MUMmer v4.0.0rc1 program (Marcais et al. 2018).

To ensure the quality of gene-based pan-genome analysis, all the assemblies were de novo annotated by the same pipeline according to those used for *A. hemsleyana* and *A. zhejiangenisis* in our previous study (Yu et al. 2023), which incorporated transcriptome, ab initio and homolog predictions were used predict protein-coding genes. RNA-seq reads were downloaded from public database (Table S13). Adapter sequences and low-quality reads were removed by fastp v0.22.0 (Chen et al. 2018). Clean reads were then aligned to each assembly using HISAT2 v2.2.1 (Kim et al. 2019), and assembled using StringTie2 v2.1.6 (Kovaka et al. 2019). The coding regions were predicted using TransDecoder v5.5.0 (http://transdecoder.github.io). For ab initio prediction, gene models were predicted using AUGUSTUS v3.3.3 (Stanke et al. 2008) and GlimmerHMM (Majoros et al. 2004). Protein sequences of *Solanum lycopersicum*, *Arabidopsis thaliana*, *A. zhejiangenisis* and *Oryza sativa* were used for homolog-based predictions. Finally, the information of repetitive sequences, transcripts, ab initio and homology-based gene predictions were integrated using MAKER v2.31.11 (Cantarel et al. 2008).

### Gene family clusters and phylogenetic analysis

OrthoFinder v2.5.2 (Emms and Kelly 2019) was used to infer a matrix of orthologous groups (gene families) among all the *Actinidia* genomes and *R. ovatum* genome. A species tree was inferred from all sets of orthogroup genes including single-copy and multi-copy orthogroups using STAG (Emms and Kelly 2018). Besides, multiple sequence alignments were performed using MUSCLE v3.8.31 (Edgar 2004) for the identified single-copy orthologous genes. A maximum likelihood (ML) phylogenetic tree was constructed using the alignments of single-copy orthologous genes with RAxML v8.2.12 (Stamatakis 2014). Species divergence time estimates were calculated using MCMCTREE in PAML (v4.9i) (Yang 2007), and calibrated using the estimated divergence times for *R. ovatum* and *A. chinensis* (53.4–99.2 Mya), *A. arguta* and *A. chinensis* (5.0–35.0 Mya) in the TimeTree database (http://www.timetree.org/). Gene family expansion or contraction was determined using CAFÉ (v3.0) (De Bie et al. 2006).

### SNP calling and phylogenetic analysis

Genome resequencing data of 114 *Actinidia* accessions from 20 species were downloaded from the NCBI Sequence Read Archive database (SRA) and Genome Sequence Archive (GSA) database (Table S2). Adapter sequences and low-quality reads were removed by fastp v0.22.0 (Chen et al. 2018). The clean reads were then mapped to the AcDH genome using BWA v0.7.17, and duplicated reads were further removed by MarkDuplicates of GATK v4.2.0 (DePristo et al. 2011). SNP calling was performed using GATK HaplotypeCaller, generating a single variant calling file (VCF). The VCF was preliminarily filtered by GATK VariantFiltration based on following criteria: QD < 2.0 || MQ < 40.0 || FS > 60.0 || SOR > 3.0 || MQRankSum < 12.5 || ReadPosRankSum < 8.0, and further filtered by VCFtools v0.1.16 (Danecek et al. 2011) with the following parameters: –maf 0.1 –minQ 30 –mac 3 –minDP 3 –max-missing 0.5.

PCA was performed by PLINK v1.90 (Purcell et al. 2007). The ancestral population structure was estimated using ADMIX TURE v1.3.0 (Alexander et al. 2009) with number of sub-populations (*K*) from 2 to 5. For phylogenetic relationship analysis, SNPs were thinned using a distance filter of interval ≥ 2000 bp. A ML tree was constructed to investigate genetic relationships within *Acitinida* by IQ-TREE (Minh et al. 2020) with 1000 rapid bootstraps. To detect selective sweeps, SNPs were subjected to XP-CLR (Chen et al. 2010) with 50-kb sliding window and 20-kb step for each chromosome. The top 5% XP-CLR values across the genome were considered be potential selected loci. *F*_ST_ was calculated in 20 kb stepping windows using VCFtools.

### Gene-based *pan*-genome construction and analysis

We constructed a gene-based pan-genome using the 15 *Actinidia* assemblies (Table S1). The core, softcore, dispensable and cloud gene sets among the 15 genomes were estimated based on gene family clustering using OrthoFinder v2.5.2 with an inflation parameter of 1.5. For each gene family, one gene was randomly chosen, and the representative protein sequences of all gene families were aligned against InterPro databases with Diamond v2.0.13.151 (Buchfink et al. 2021). GO terms were assigned according to the InterPro classification. GO enrichment analysis with gene families was performed using Fisher’s exact test with an adjusted *P*-value (*q* value) of < 0.05.

To analyze the length and exon numbers of core, softcore and dispensable genes, all core, softcore and dispensable genes of *A. arguta*, *A. eriantha* and AcDH were used. Nucleotide diversity (π) was calculated by VCFtools. The RNA-seq data used for calculated the expression levels of core, softcore and dispensable genes were obtained from public databases (Table S13). Raw reads were filtered to remove adapter and low-quality sequences by fastp. Cleaned reads were mapped to the corresponding assembly using HISAT2 v2.2.1 (Kim et al. 2019), and gene expression levels were calculated using StringTie v2.1.6 (Kovaka et al. 2019). To find the gene families related to sex determination, gene families present in each male assembly but absent in any female assembly, that is, specific to male accessions were analyzed.

### SVs identification and graph-based *pan*-genome construction

TGS data was downloaded from public databases (Table S8). TGS data and genome assemblies were used to identify SVs against AcDH using PanPop (Zheng et al. 2024), a sequence-aware SV merging and processing pipeline, with default parameters. For *A. zhejiangenisis,* which contained genome information of two species, only TGS data was used for SV calling. Besides, SVs were investigated using only TGS data for another two *Actinidia* accessions with public TGS data but not assemblies (*A. chinensis* 'H0809' and *A. eriantha* 'Blank'). To enhance accuracy of SVs, five SV callers, sniffles, cuteSV, svim, pbsv, and Assemblytics were used, and SVs supported by at least two callers were retained. The merged nonredundant variations in VCF format were used to construct the graph-based pan-genome by vg toolkit v1.50.1 (Garrison et al. 2018).

To identify population-scale SVs, the clean short reads of the 112 accessions (Table S2, two individuals (*A. chinensis*_5 and *A. chinensis*_6) with single-end sequencing data were removed here) were mapped to the graph-based genome by giraffe and SVs were then filtered and merged using PanPop with default parameters. Those SVs were further filtered by PLINK with the following parameters: –geno 0.5 –maf 0.05. PCA analysis was performed via PLINK. To build a phylogenic tree with SVs, *P*-distance matrix based VCF was generated by VCF2Dis (https://github.com/BGI-shenzhen/VCF2Dis), and then used to construct a neighbor-joining tree by FastME 2.0 (Lefort et al. 2015). To identify SV hotspot regions, we calculated the distribution of SVs for each 100 kb window (with a 20 kb step size) along each chromosome. The top 5% of all windows with the highest frequency of variations were selected, and then merged as SV hotspot regions. SVs in intergenic, upstream, downstream, intron and exon regions were annotated using ANNOVAR (Wang et al. 2010), and the number was calculated in each accession. To analyze the frequencies of SVs, the genotyped SVs of each group were obtained by VCFtools, and the frequencies of SVs were then calculated, respectively. RNA-seq data used here was obtained from public databases (Table S13). Gene expression levels were calculated by StringTie, and read counts were estimated by prepDE.py script of StringTie. Differentially expressed genes were identified by DESeq2 (Love et al. 2014).

### RGA identification and *pan*-RGA analysis

The deduced protein sequences of the annotated genes of all 15 assemblies were used to identify RGAs via the RGAugury pipeline (Li et al. 2016). The identified RGA candidates included NBS-encoding proteins, RLKs, RLPs and TM-CCs. The syntenic gene pairs between species were identified using JCVI v.1.3.8, a Python version of MCscan (Tang et al. 2008). IDs of NBS proteins were predicted using HMMER 3.1b2 (https://github.com/EddyRivasLab/hmmer). To analyze the expression pattern of paired NLR or clustered NBS genes in disease response, RNA-seq data of *A. chinensis*, *A. eriantha* and *A. arguta* under *Psa* treatment was downloaded (Table S13), and clean reads were mapped to AcDH, *A. eriantha* ‘Midao31’ and *A. arguta* ‘M1’ using HISAT2, respectively. Gene expression levels were then calculated by StringTie. Gene family clustering was performed with all RGAs by OrthoFinder and core, softcore, dispensable and cloud RGA gene sets were subsequently estimated.

## Supplementary Information

Additional file 1: Fig. S1. GO enrichment analysis of genes in the highly differentiated regions between LG and MG by *F*_ST_ analysis. Fig. S2. Gene number in each gene family of the pan-genome. Fig. S3. Expression levels of core, softcore and dispensable genes in different species. (a) Expression levels of genes in the fruits of *A. chinensis* ‘Donghong’ at different days after flowering (DAF). (b) Expression levels of genes in different tissues from *A. arguta* ‘M1’. Fig. S4. GO enrichment analysis of core genes from the gene-based kiwifruit pan-genome. Fig. S5. Phylogenic tree (a) and principal component analysis (b) based on SVs from 112 *Actinidi*a accessions. Azh: *A. zhejiangensis*. Fig. S6. GO enrichment analysis of genes in SV hotspot regions. Fig. S7. GO enrichment analysis of genes overlapping with SVs with high frequency (> 0.5) in LG but absent in MG. Fig. S8. Number of RLK, RLP and TM-CC genes on each chromosome. Fig. S9. NBS gene cluster size and frequency in each assembly. Fig. S10. Word cloud of integrated domains of NBS genes in *Actinidia*. Fig. S11. Venn diagram of NBS integrated domains of selected species. Fig. S12. Proportion of core, softcore, dispensable and cloud gene family from pan-RGA in each assembly. Fig. S13. Phylogenic tree of *Actinidia* species based on single-copy orthologous genes.

Additional file 2: Table S1. All the *Actinidia* assemblies used in this study. Table S2. The genome resequencing data used in this study. Table S3. Highly differentiated regions between LG and MG. Table S4. Selected regions of MG by XP-CLR analysis. Table S5. Genes in both the HDR and XP–CLR selected region. Table S6. Gene families specific to LG or MG. Table S7. Gene families specific to male accessions. Table S8. The genome assemblies and third-generation sequencing data used for graph-based pan-genome construction. Table S9. SV hotspot regions in *Actinidia*. Table S10. Details of SVs involved in this study. Table S11. The genes overlapping with MG1- or MG2-favored SVs. Table S12. The RGA gene families absent in *A. chinensis* and MG. Table S13. RNA-seq data used in this study.

## Data Availability

The pan-genome datasets and all the identified RGAs in *Actinidia* can be found at https://figshare.com/articles/dataset/KiPan/26075293.

## Abbreviations

*A. arguta*: *Actinidia arguta*
*A. chinensis*: *Actinidia chinensis*
*A. eriantha*: *Actinidia eriantha*
*A. hemsleyana*: *Actinidia hemsleyana*
*A. latifolia*: *Actinidia latifolia*
*A. ploygama*: *Actinidia polygama*
*A. rufa*: *Actinidia rufa*
*A. valvata*: *Actinidia valvata*
*A. zhejiangensis*: *Actinidia zhejiangensis*
AcDH: *A. chinensis* cv. Donghong
AsA: Ascorbic acid
DEG: Differentially expressed gene
FPKM: Fragments per kilobase of exon model per million mapped fragments
FST: Fixation index
GO: Gene ontology
GSA: Genome Sequence Archive
GSH: Glutathione
HDR: Highly differentiated region
ID: Integrated domain
ILS: Incomplete lineage sorting
LG: Leiocarpae group
MG: Maculatae group
ML: Maximum likelihood
Mya: Million years ago
NAP1: Nck-associated protein 1
NCBI: National Center for Biotechnology Information
NBS: Nucleotide-binding site
NLR: Nucleotide-binding leucine-rich repeat
PAV: Presence/absence variant
Psa: Pseudomonas syringae pv. Actinidiae
PCA: Principal component analysis
QTL: Quantitative trait locus
RGA: Resistance gene analog
RLK: Receptor-like protein kinase
RLP: Receptor-like protein
SNP: Single nucleotide polymorphism
SRA: Sequence Read Archive database
SV: Structural variation
T2T: Telomere-to-telomere
TM-CC: Protein with both transmembrane domain and coiled-coil domain
TGS: Third-generation sequencing
VCF: Variant calling file π Nucleotide diversity
