# Correction: Super pan-genome reveals extensive genomic variations associated with phenotypic divergence in *Actinidia*

**DOI:** 10.1186/s43897-025-00154-2

**Published:** 2025-03-19

**Authors:** Xiaofen Yu, Minghao Qu, Pan Wu, Miao Zhou, Enhui Lai, Huan Liu, Sumin Guo, Shan Li, Xiaohong Yao, Lei Gao

**Affiliations:** 1 State Key Laboratory of Plant Diversity and Specialty Crops, Wuhan Botanical Garden, Chinese Academy of Sciences, Wuhan, Hubei 430074 China; 2 Hubei Hongshan Laboratory, Wuhan, Hubei 430070 China; 3 University of Chinese Academy of Sciences, Beijing, 100049 China; 4 Bioinformatics Center, College of Plant Science and Technology, Beijing University of Agriculture, Beijing, China

**Correction: Mol Hortic 5, 4 (2025)**

**https://doi.org/10.1186/s43897-024–00123-1**

Following publication of the original article (Yu et al. 2025), the authors reported an error in authorship, where Xiaohong Yao should be marked as a corresponding author.

The full list of authors is corrected from:

Xiaofen Yu^1,2^, Minghao Qu^1,3^, Pan Wu^1^, Miao Zhou^1,3^, Enhui Lai^1,3^, Huan Liu^1,4^, Sumin Guo^1^, Shan Li^1^, Xiaohong Yao^1^ and Lei Gao^1,2*^

*Correspondence:

Lei Gao

leigao@wbgcas.cn

To:

Xiaofen Yu^1,2^, Minghao Qu^1,3^, Pan Wu^1^, Miao Zhou^1,3^, Enhui Lai^1,3^, Huan Liu^1,4^, Sumin Guo^1^, Shan Li^1^, Xiaohong Yao^1*^ and Lei Gao^1,2*^

*Correspondence:

Xiaohong Yao

yaox@wbgcas.cn

Lei Gao

leigao@wbgcas.cn

The original article (Yu et al. 2025) has been updated.
