# Supplementary material for: Super pan-genome reveals extensive genomic variations associated with phenotypic divergence in Actinidia
Source: Mol Hortic. 2025 Jan 24;5:4. doi: 10.1186/s43897-024-00123-1 (PMC11758757; doi:10.1186/s43897-024-00123-1)
Supplement: Supplementary file 1 — Additional file 1: Fig. S1. GO enrichment analysis of genes in the highly differentiated regions between LG and MG by FST analysis. Fig. S2. Gene number in each gene family of the pan-genome. Fig. S3. Expression levels of core, softcore and dispensable genes in different species. (a) Expression levels of genes in the fruits of A. chinensis ‘Donghong’ at different days after flowering (DAF). (b) Expression levels of genes in different tissues from A. arguta ‘M1’. Fig. S4. GO enrichment analysis of core genes from the gene-based kiwifruit pan-genome. Fig. S5. Phylogenic tree (a) and principal component analysis (b) based on SVs from 112 Actinidia accessions. Azh: A. zhejiangensis. Fig. S6. GO enrichment analysis of genes in SV hotspot regions. Fig. S7. GO enrichment analysis of genes overlapping with SVs with high frequency (> 0.5) in LG but absent in MG. Fig. S8. Number of RLK, RLP and TM-CC genes on each chromosome. Fig. S9. NBS gene cluster size and frequency in each assembly. Fig. S10. Word cloud of integrated domains of NBS genes in Actinidia. Fig. S11. Venn diagram of NBS integrated domains of selected species. Fig. S12. Proportion of core, softcore, dispensable and cloud gene family from pan-RGA in each assembly. Fig. S13. Phylogenic tree of Actinidia species based on single-copy orthologous genes. [file 43897_2024_123_MOESM1_ESM.docx]

**Super pan-genome reveals extensive genomic variations associated with phenotypic divergence in *Actinidia***

Xiaofen Yu^1,2^, Minghao Qu^1,3^, Pan Wu^1^, Miao Zhou^1,3^, Enhui Lai^1,3^, Huan Liu^1,4^, Sumin Guo^1^, Shan Li^1^, Xiaohong Yao^1,*^, Lei Gao^1,2,*^

^1^ State Key Laboratory of Plant Diversity and Specialty Crops, Wuhan Botanical Garden, Chinese Academy of Sciences, Wuhan, Hubei 430074, China

^2^ Hubei Hongshan Laboratory, Wuhan, Hubei 430070, China

^3^ University of Chinese Academy of Sciences, Beijing 100049, China

^4^ Bioinformatics Center, College of Plant Science and Technology, Beijing University of Agriculture, Beijing, China

*Correspondence (Tel/fax +86-27-87700860; emails: [yaox@wbgcas.cn](mailto:yaox@wbgcas.cn) [XY], [leigao@wbgcas.cn](mailto:leigao@wbgcas.cn) [LG])


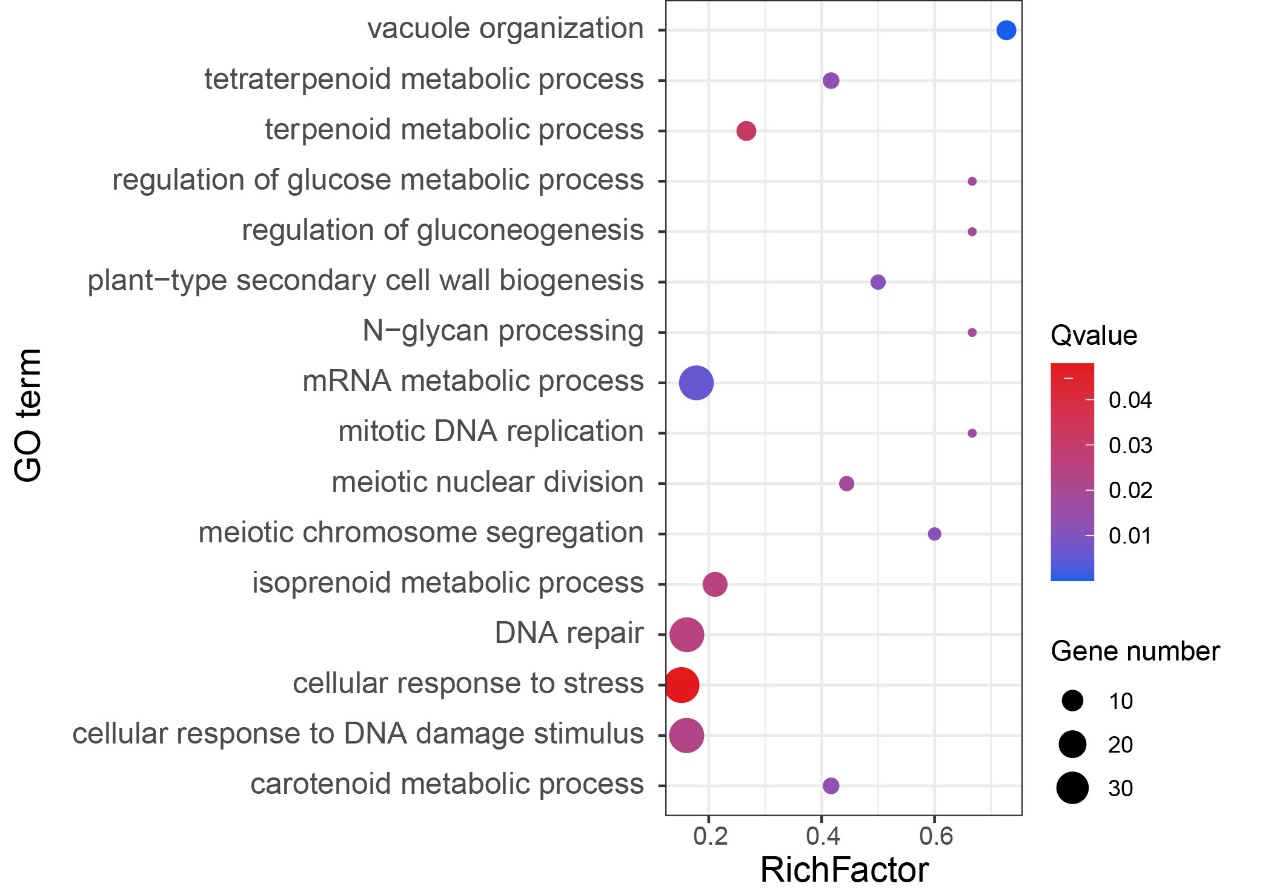


**Fig. S1**. GO enrichment analysis of genes in the highly differentiated regions between LG and MG by *F*_ST_ analysis.


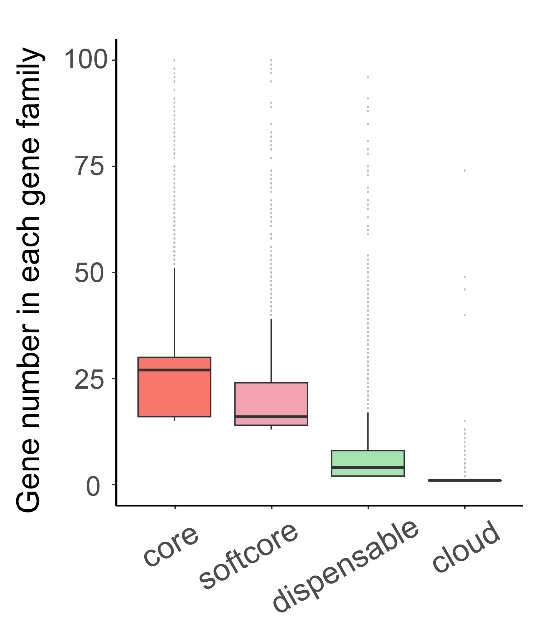


**Fig. S2**. Gene number in each gene family of the pan-genome.


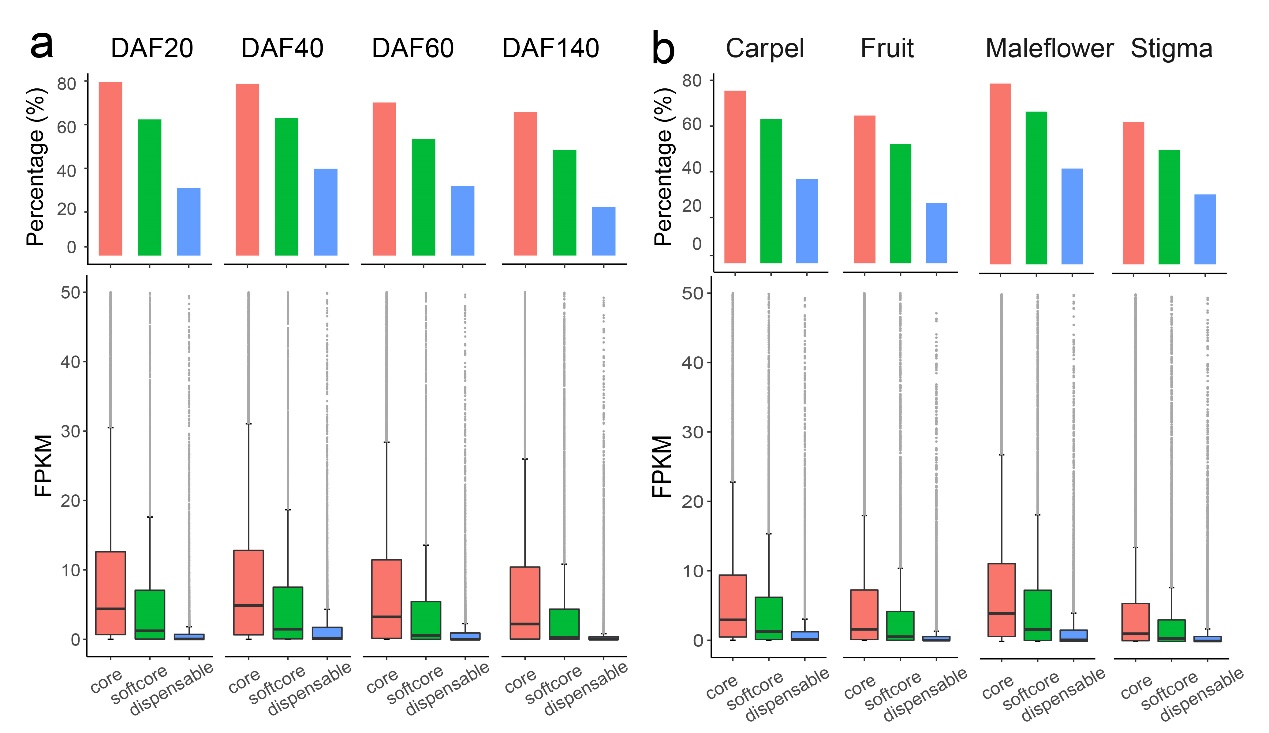


**Fig. S3**. Expression levels of core, softcore and dispensable genes in different species. (**a**) Expression levels of genes in the fruits of *A. chinensis* ‘Donghong’ at different days after flowering (DAF). (**b**) Expression levels of genes in different tissues from *A. arguta* ‘M1’.


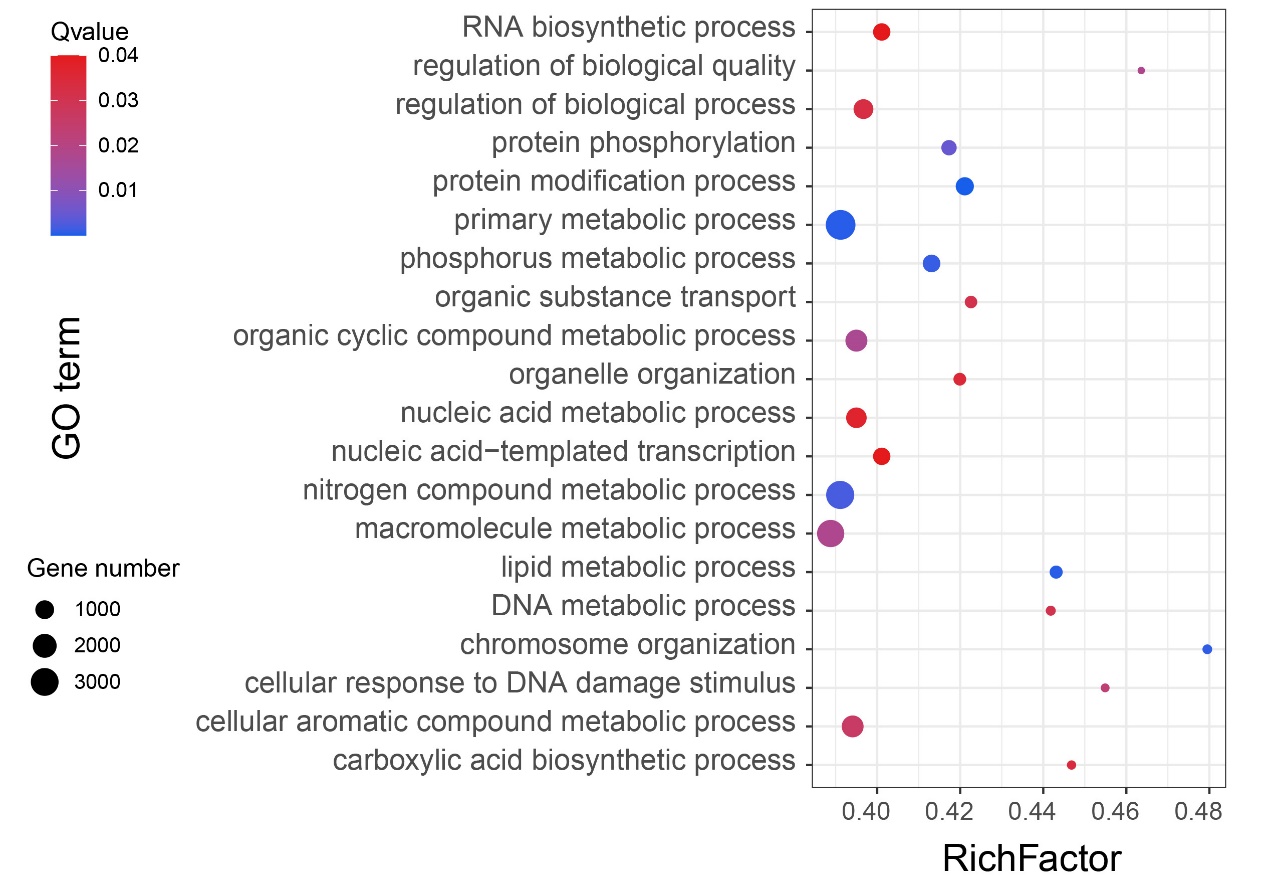


**Fig. S4**. GO enrichment analysis of core genes from the gene-based kiwifruit pan-genome.


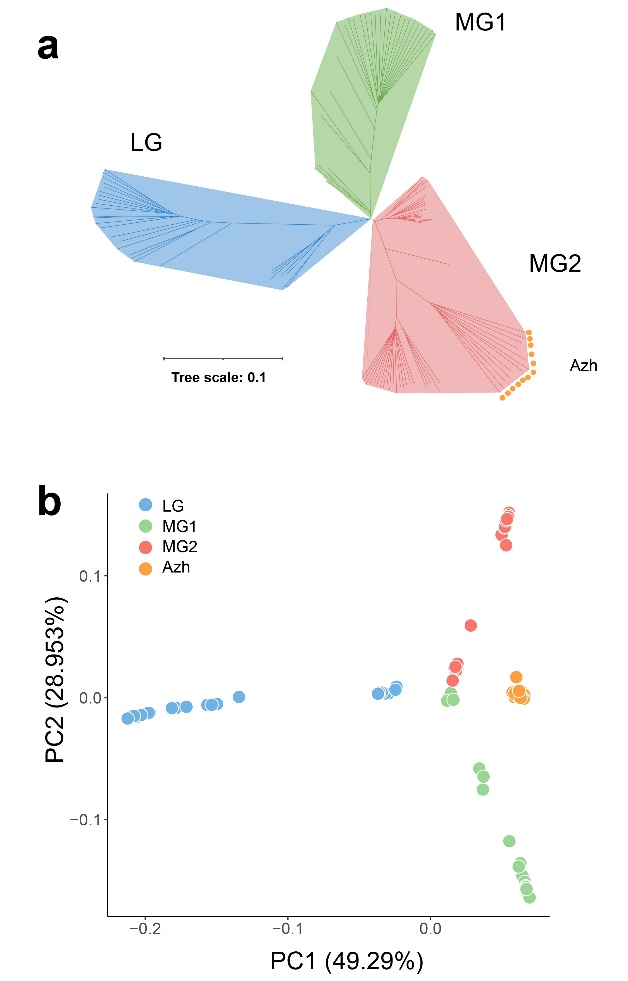


**Fig. S5**. Phylogenic tree (a) and principal component analysis (b) based on SVs from 112 *Actinidi*a accessions. Azh: *A. zhejiangensis*.


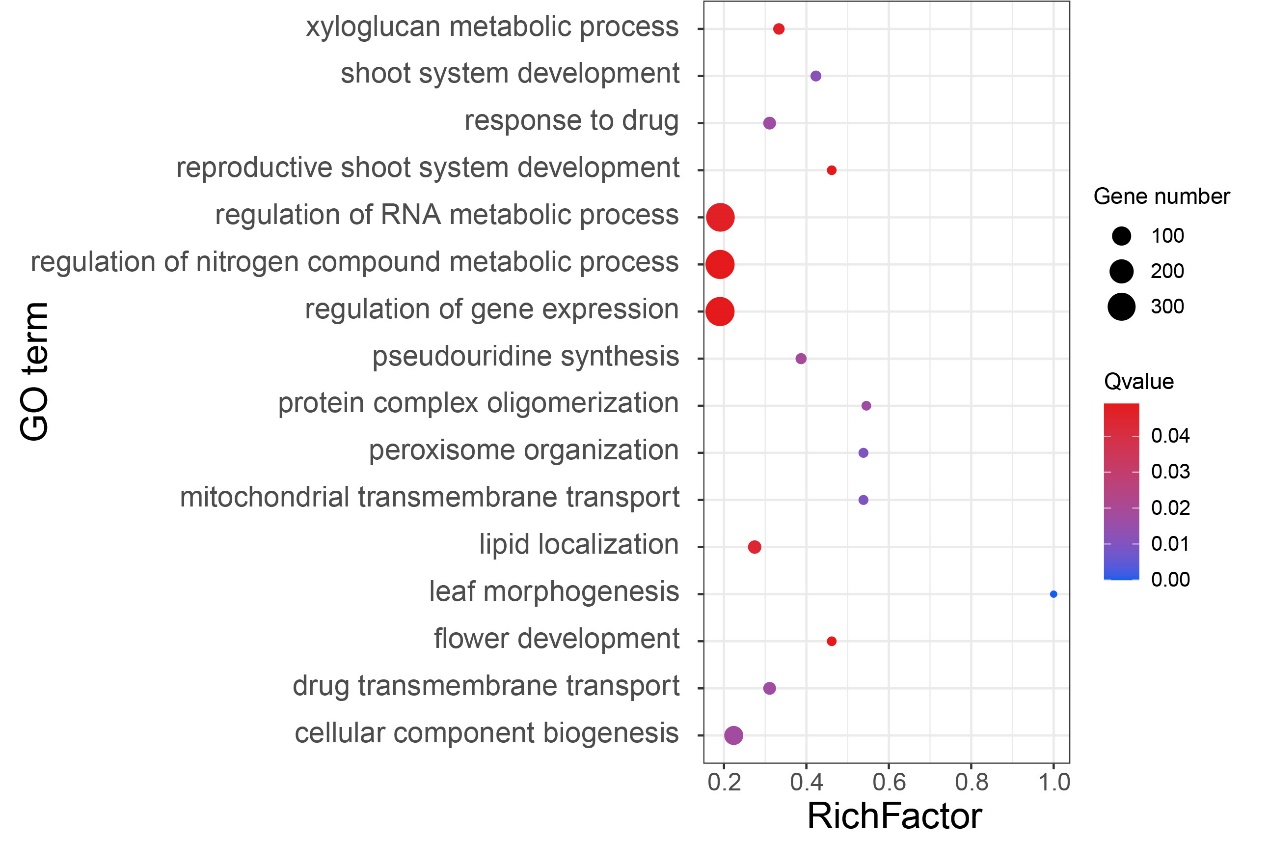


**Fig. S6**. GO enrichment analysis of genes in SV hotspot regions.


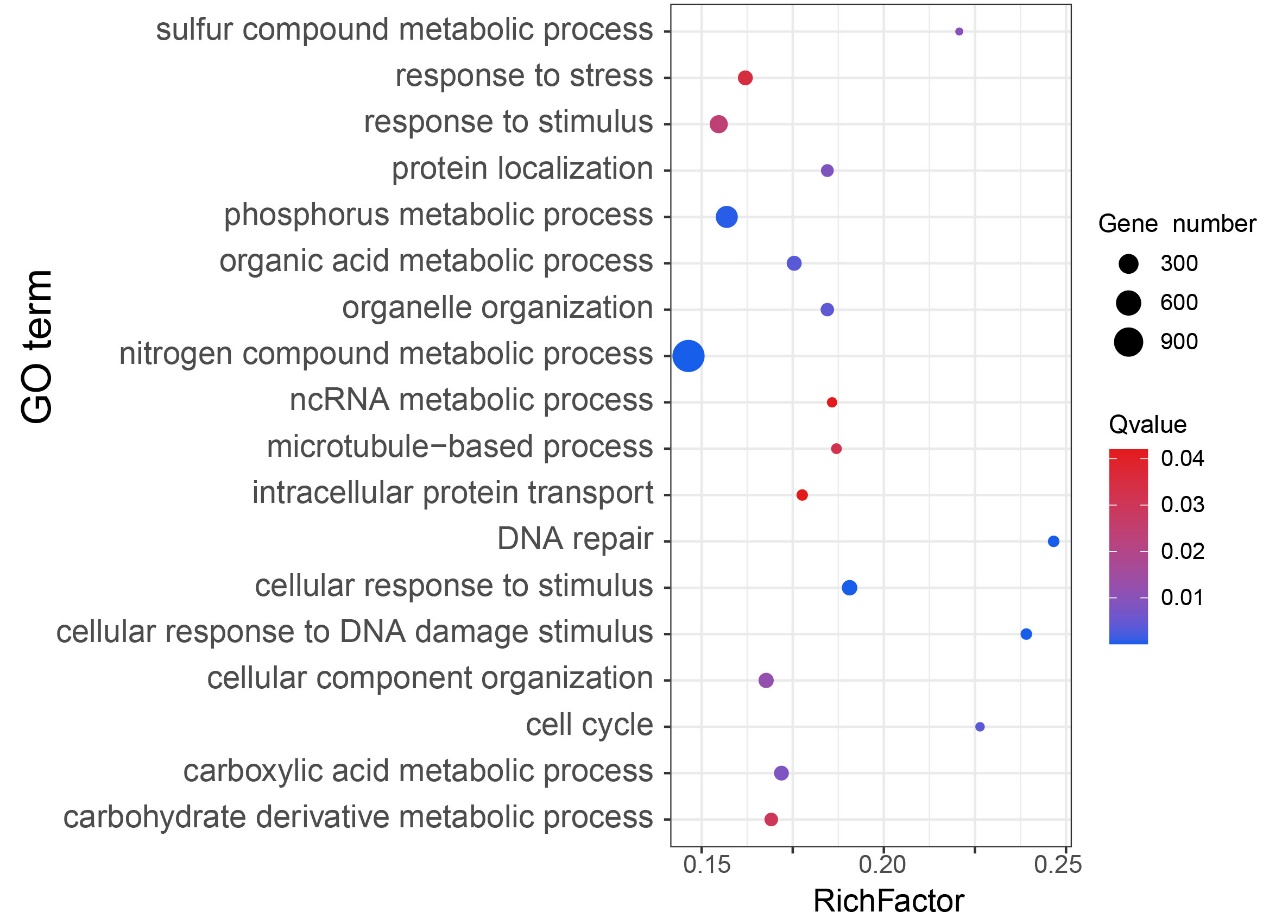


**Fig. S7**. GO enrichment analysis of genes overlapping with SVs with high frequency (>0.5) in LG but absent in MG.


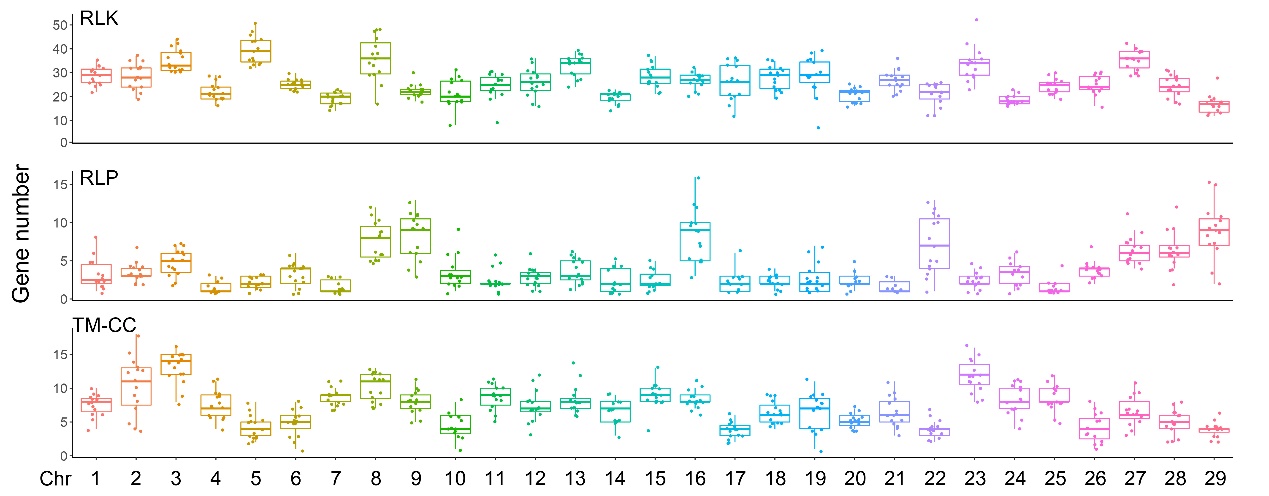


**Fig. S8**. Number of RLK, RLP and TM-CC genes on each chromosome.


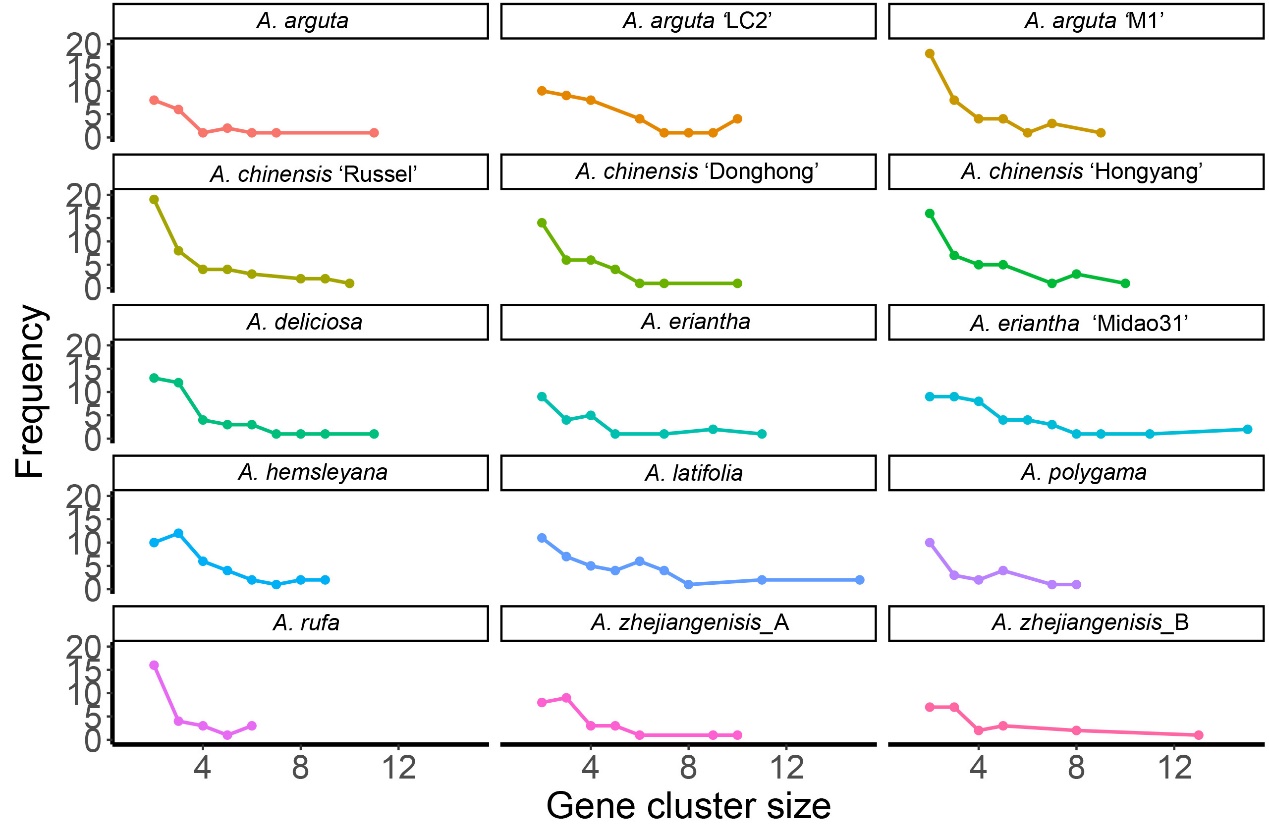


**Fig. S9**. NBS gene cluster size and frequency in each assembly.


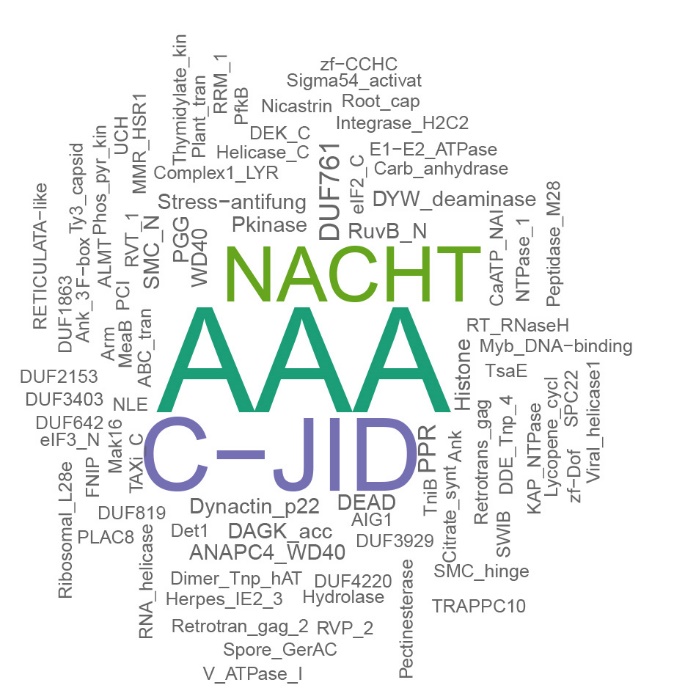


**Fig. S10**. Word cloud of integrated domains of NBS genes in *Actinidia*.


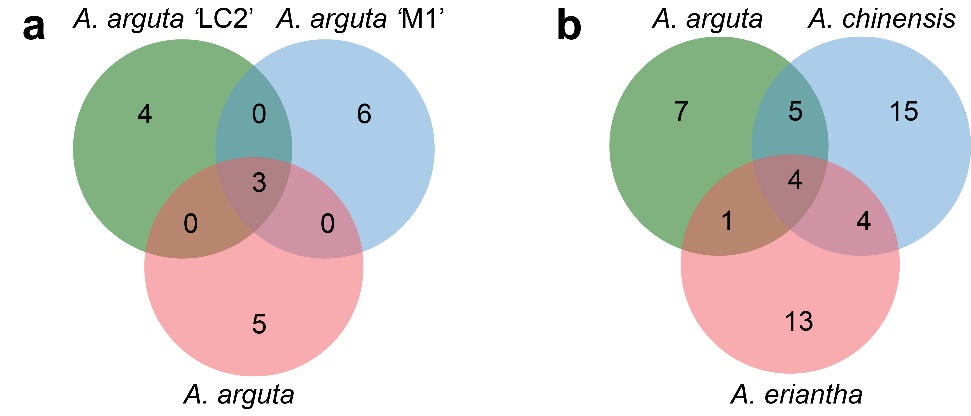


**Fig. S11**. Venn diagram of integrated domains of selected species.


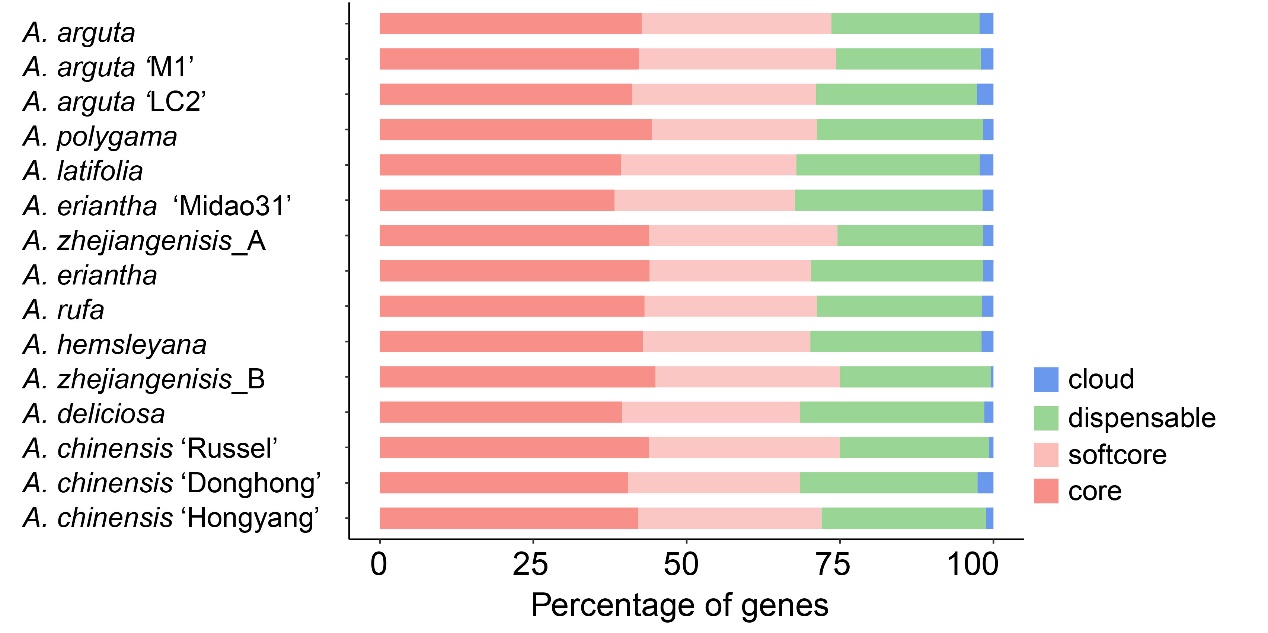


**Fig. S12**. Proportion of core, softcore, dispensable and cloud gene family from pan-RGA in each assembly.

**
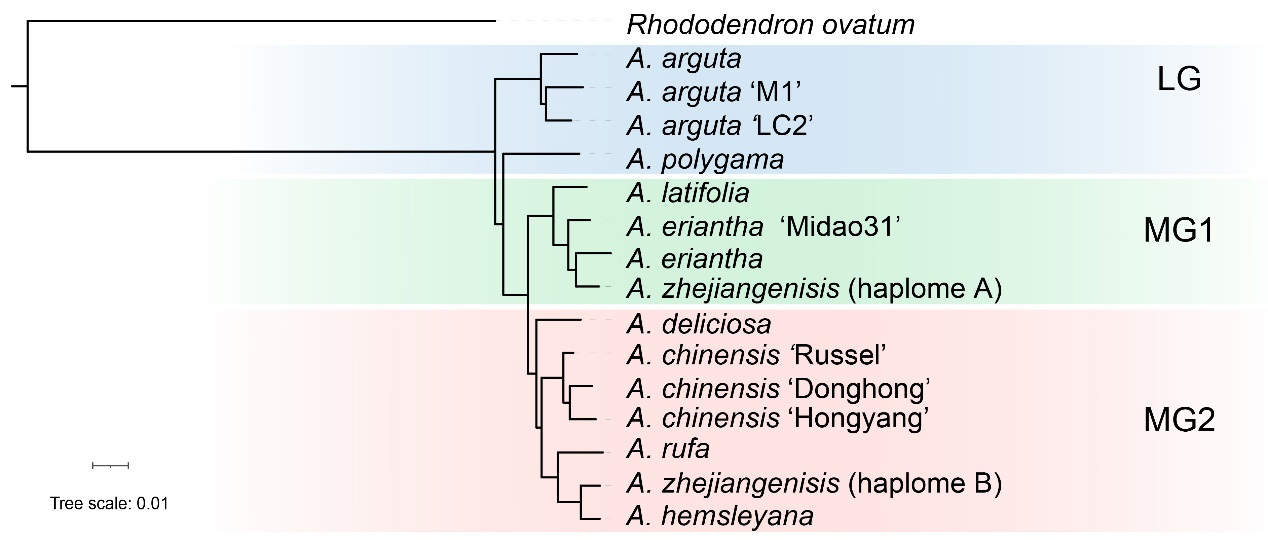
**

**Fig. S13**. Phylogenic tree of *Actinidia* species based on single-copy orthologous genes.
